# Does corporate governance mechanism deter earnings management and enhance readability of annual reports?

**DOI:** 10.1371/journal.pone.0311543

**Published:** 2025-02-14

**Authors:** Dong Zheng, Rajib Ali, Zhao Feifei, Muhammad Shaique

**Affiliations:** 1 Qingdao University Business College, Qingdao, Shandong, People’s Republic of China; 2 Department of Business Administration, Hands Institute of Development Studies, Karachi, Pakistan; 3 Shandong Youth University of Political Science, School of Economics and Management, Jinan, Shandong, China; 4 Department of Business Administration, Sukkur IBA University, Sukkur, Pakistan; An-Najah National University, STATE OF PALESTINE

## Abstract

This study aims to know the impact of earnings management (accrual, real and total) and corporate governance mechanisms on the readability of annual reports. Additionally, the study also seeks to know the moderating impact of corporate governance mechanisms between earnings management (accruals, real and total) and readability of annual reports. The sample of the study consists 250 listed firms of Pakistan Stock Exchange (PSX) for the period of 2014–2022. The hypotheses are tested using System GMM technique. The results of the study suggest that earnings management (accruals, real and total) has negative and significant impact on readability of annual reports. However, all four corporate governance mechanics have a significantly positive impact on readability of annual reports. Additionally, corporate governance mechanics significantly moderates the relationship between earnings management (accruals, real and total) and the readability of annual reports. This study has practical implications for regulators, investors, and firms. The findings of the study may suggest to the Security Exchange Commission Pakistan that authorities improve readability by requiring companies to use clear, understandable language and include appropriate information in annual reports. Firms listed on PSX need to produce more readable annual reports to make information more concise and clear, using simple and short sentences, and familiar words.

## 1. Introduction

The annual report published by the end of the year consists of financial and non-financial (description, images, and charts) information [1]. The ultimate objective is to communicate with the investors, lenders, and other stakeholders in a transparent manner [2] for relevant decision making and resource allocation. However, sometimes the text is written in complex language which becomes difficult to comprehend. This language complexity is called readability of annual reports (RAR), that refers to “how easily the audience can comprehend the information available in the annual reports which is crucial for informed decision-making”. The intension is to protect unfavorable practices of corporate behavior [3] or entrenchment behavior of management [4]. This language complexity hampers stakeholders from leveraging the information related to the performance of the company. Therefore, it is vital to consider the factors that influence the readability of annual reports (RAR) especially in Pakistan, where literacy rate is low and the comprehension of English language is poor as well [5]. This lack of readability also indicates the existence of various inefficiencies, as well as elevates the probabilities of information asymmetry.

Another motivation behind this research is the ongoing concerns over earnings management (EM) in developed and developing countries. Earnings management is the process of manipulating firm’s numbers in the financial statements to present a more favorable view of its financial position. This manipulation often leads to complex and ambiguous language in the annual report, which creates information asymmetry and making it difficult for audience to measure actual financial position of the company [6].

Firm management often seeks to publish only positive results, which can lead to information asymmetry between managers and shareholders due to which conflicts of interest arises between them. As per the agency theory, these conflicts of interest arise due to the managers may prioritize their interests over those of the shareholders [7]. Earnings management worsens this issue by concealing the actual operating performance, thus decreasing the levels of transparency and accountability [8]. As a result, it is significant to reduce agency cost through higher disclosure quality and by producing easy-to-read annual reports [2].

Corporate governance plays an essential role in controlling and monitoring these language issues. Active corporate governance mechanisms, such as board independence and audit quality, help ensure that financial reports are credibility and transparent [9]. These mechanisms can solve the problem of reports language, reduce agency costs and information asymmetry and, therefore, the goals of managers and shareholders are aligned [10]. This alignment motivates managers to work in the stakeholders’ favor especially owing to the emphasis on shareholder [11].

The existing phenomenon of readability of annual reports still gives management the room to manipulate impressions of annual reports to obscure stakeholders. The increased difficulty in readability has negatively influenced the reason of publishing annual reports. It is crucial to study the reason behind this management exploitation. The aim of the study is to examine the impact of earnings management on the readability of annual reports. Specifically, Fields, Lys [12] and Zang [13] suggested that examining the individual impact of both accrual earnings management (ABEM) and real activity earnings management (RBEM) strategies cannot give the overall impact of EM. To best of our knowledge, there is paucity of research available on the combined impact of earnings management (real and accruals) on readability of annual reports. Moreover, this study also seeks to know the impact of corporate governance mechanisms on the readability of annual reports and the moderating role of corporate governance mechanisms between earnings management and readability of annual reports.

This study contributes to the existing body of knowledge and makes the following contributions to the literature. First, most of the studies on readability of annual reports are conducted in English-speaking countries like the US. This area is relatively less researched in Pakistan where English is not the first language and literacy rate is very poor [5]. Due to the cultural, political, and economic setting in the country with a dynamic corporate governance environment [14], the effect of the earnings management on report readability offers an interesting case to analyze on the backdrop of Pakistan. Hence, based on the context of Pakistan, this study drives awareness about the enhanced report readability for non-English-speaking and developing nations, leading to better financial reporting. The present study developed the readability indices (Fog index and Flesch-Kincaid grade level) of Pakistani firms’ annual reports, which could be an essential orientation for the future researches in readability prospects in Pakistan. Secondly, there is a paucity of research on the moderating role of corporate governance mechanisms between readability of annual reports and EM. Hence, the present study seeks to know the moderating role of corporate governance mechanisms. The primary objective of corporate governance is to eliminate fraudulent activities and to reduce information asymmetry in the firms. In the presence of strong corporate governance, firms will engage in the earnings management may avoid complex language. Thirdly, the regulatory authority is taking steps to facilitate investors by providing financial education; thus, study findings have implications for the regulators to encounter comprehensive issues for increasing financial inclusion. In short, the study results may recommend that the authorities elementary language and provide the relevancy. Finally, this study extends the literature on the readability of annual reports, earnings management, and corporate governance mechanisms.

The study results confirm that firms produce obscure reports and intentionally make annual reports difficult to understand to overshadow managers’ opportunistic behavior. Further, all four corporate governance mechanics (board independence, audit committee size, audit quality and female directors) have a significantly positive impact on readability of annual reports. Each corporate governance variable is crucial in providing more readable annual reports to minimize information asymmetry and to reduce agency problems. Additionally, corporate governance variables play an essential role in as moderating variable between earnings management and readability of annual reports.

## 2. Literature review and hypothesis development

### 2.1. Earnings management and readability of annual reports

Previous studies on annual reports readability have found that the reports are difficult to understand and comprehend. Li [15] argues that when firms make losses, they produce less readable reports. Additionally, he stated that firms with persistent earnings make more readable reports (less fog index). According to Biddle [16], better financial reporting increases investment efficiency and reduces the amount that deviates from the level of investment that was anticipated. Moreover, in an effort to conceal from investors any negative future performance, managers restrict the amount of disclosure items in financial reports [15]. It is clear from this that managers may use readability as a useful tool to provide information about the company [3].

The firms disclosure quality can be improved with a higher degree of readability of annual reports, which may reduce the information asymmetry [17]. Moreover, Bloomfield [18] criticized the negative association between the firms’ readability and the firm’s performance, but it is still ambiguous about the reason for this relationship. Either manager provides complex language in narrative disclosures to hide poor performance (obfuscation hypothesis) or simply because adverse news is more challenging to communicate. The obfuscation hypothesis is defined as writing reports in style to blur the intended message which is the manipulative behavior of the managers [19]. Therefore, it is vital to examine the narrative accounting disclosures to know whether managers purposefully make more complex information to change readers’ perceptions.

In the literature, Arora and Chauhan [17] found that firm engage in earnings management have a less readable annual reports. It means that firms use complex language in annual reports while they are engaged in EM. Furthermore, Alm El-Din, El-Awam [20] found that to conceal financial manipulation, companies that use earnings management produce less legible annual reports in the context of European countries. Additionally, Lo, Ramos [6] and Ajina, Laouiti [2] argued that earnings management negatively impact the readability of annual reports in the context of US. In short, firms engaged in accrual based earnings management (ABEM) and real activity base (RBEM) make less readable annual reports to hide financial manipulation. In comparison to the negative impact of real earnings management on readability of annual reports, Seifzadeh, Salehi [21] found that RBEM has a positive and significant influence on readability in the context of Iran. Hence, it can be assumed that firms provide more readable annual reports when firms engaged in RBEM but less readable reports when firms engaged in ABEM. Due to these contradicting results in different contexts for the real EM, it seems practically significant to see the language complexities of the annual reports for earnings management in Pakistan. The present study may argue that the managers make complex language annual reports to hide the earnings management from investors and other stakeholders. Additionally, there is a dearth of research on readability and earnings management for developing nations like Pakistan. When it comes to the institutional framework, Pakistan is less regulated than developed nations like the US in terms of rules and regulations being enforced. Therefore, the following hypothesis is proposed:

**Hypothesis 1a:** ABEM has a negative impact on the readability of annual reports.
**Hypothesis 1b:** RBEM has a negative impact on the readability of annual reports.

This study also seeks to know the combined impact of ABEM and RBEM on readability of annual reports. As the combined effect of earnings management is studied by Bhutto, Shaique [22] on stock returns. They found that the combined effect of earnings management negatively influences the stock return. As per the author’s best knowledge, little or paucity research has been done on the combined impact of earnings management on the readability of annual reports in Pakistan. Hence following hypothesis is proposed:

**Hypothesis 1c:** Combined effect of RBEM and RBEM has a negative impact on the readability of annual reports.

### 2.2. Corporate governance mechanisms and readability of annual reports

#### 2.2.1. Independent board of directors (IBD)

The minority shareholders’ rights can be ensured by having independent directors on board. Therefore, permitting minority shareholder representatives on the board could significantly reduce agency conflict [23]. In addition, García and Herrero [24] supported the role of IBDs is to increase stakeholders’ confidence by enhancing transparency and consistency in the disclosers. This study argued that IBD is considered a good method to decrease impression management and improve narrative disclosure to make easy-to-read annual reports.

Tessema [25] argued that a larger proportion of IBD is more successful in controlling the opportunistic behavior of managers. Similarly, Goh, Lee [26] assessed the impact of IBD on information asymmetry, and they proposed that IBD tends to decrease information asymmetry because of two essential mechanisms, greater analyst coverage and voluntary disclosure. From the available literature, it can be presumed that the independent directors work for the interests of the investors and other stakeholders. They want the information shared by firms through annual reports comprehended by the stakeholders for decision making. Hence study proposes the hypothesis as:

**Hypothesis 2a:** Higher proportion of IBD has a positive impact on the readability of annual reports.

#### 2.2.2. Audit committee size

In the corporate governance structure, the audit committee (hereafter, AC) is mainly accountable for overseeing the financial reporting process, ensuring the credibility of disclosures and help in retaining the trust of stakeholders. The main role of AC is to monitor financial reports, manage risk, ensure internal controls, and look after internal and external auditors [27]. However, Hoitash, Hoitash [28] argue that the ACZ is an important mechanism for creating transparency in financial reporting quality. The committee ratifies the adequacy and transparency of information disseminated by the managers to investors in annual reports to decrease asymmetric information between firms and stakeholders.

To effectively monitor the actions of managers, the AC must have sufficient strength with sufficient resources to carry out its responsibilities [29, 30]. For instance, Cormier, Ledoux [31] examined the negative relationship between ACZ and information asymmetric. It refers that ACZ minimizes the information asymmetry between management and stakeholders. Persons [32] provided the empirical evidence that ACZ leads to create more voluntary disclosures. In general, it can be presumed that AC favors the stakeholders’ interest by monitoring the financial reports to disseminate transparent information with stakeholders. The primary source of information for stakeholders is the annual reports, which should be readable and understandable to extract relevant information and helps in reducing information asymmetry. Therefore, the study proposes the hypothesis as:

**Hypothesis 2b:** ACZ size has a positive impact on readability of annual reports.

#### 2.2.3. Audit quality

Previous literature demonstrates that higher audit quality increases investment efficiency [33], enhance the quality of voluntary discloser [34], increase transparency and compliance [35, 36], enhance internal control quality [37], and better earnings predictability [38].

From the literature, the present study came to know that audit quality can be measured through three proxies such as audit firm size (Big-4), audit fees, and tenure of the auditor [33, 34, 39]. Shahzad, Rehman [33] empirically examined that higher AQ and good reporting quality tend to increase investment efficiency which decreases the information asymmetry. Similarly, Clinch, Stokes [39] added that higher AQ plays a vital role in the financial reporting quality by disseminating the symmetric information among stakeholders. Agyei-Mensah [34] documented that audit size, and AC effectiveness are vital in making more transparent disclosure. Additionally, Deshmukh and Zhao [40] Big-4 may enhance the readability by providing easy-to-read annual reports. However, a proposition can be drawn from the cited literature that higher AQ improves the readability of annual reports.

**Hypothesis 2c:** AQ has a positive impact on readability of annual reports.

#### 2.2.4. Female board of directors (FD)

The board diversity can bring different expertise and experience, providing the supporting mechanisms to reduce information asymmetry and agency conflict [41]. Particularly, female presence in the board provides more effective monitoring control and is more likely to raise questions that male directors would not about firms decisions [42]. As a result, the inclusion of women on boards increases a firm’s performance and efficiency [41].

The theories of economics, sociology, and psychology by establishing certain behavioral differences between men and women state that women can affect the nature and dynamics of board deliberations, which may positively influence the information environment of the firm [43]. For example, board gender diversity may bring positivity in the information atmosphere, more rigorous discussions in business decisions, and to share more transparent and understandable information with stakeholders. As a result, the information will be more transparent and decrease information asymmetry [44, 45].

Furthermore, it is well known that in comparison to males, females follow more ethical values, take certain decisions, and show less overconfidence. Women directors, as a result of these traits, expect accurate and improved financial reporting, better audit commitment, and are less likely to manage reported earnings to minimize legal and reputational risk [43, 44]. Gul, Hutchinson [46] argued that gender diversity favors better disclosure quality, firm information, and lower information asymmetry. Moreover, Gul, Srinidhi [44] also support that board gender diversity is crucial in exchanging transparent and accurate information. Therefore, our study drawn the following proposition:

**Hypothesis 2D:** Higher FD proportion on board has a positive impact on the readability of annual reports.

### 2.3. The moderating role of corporate governance between the earnings management and readability of annual reports

Corporate governance has played a substantial role in strengthening the transparency of annual disclosure and limiting the earnings management by introducing an active monitoring and control system [26, 31, 34]. In this direction, many studies have tested the association between corporate governance mechanism and earnings management. For example, corporate governance mechanisms (i.e. IBD, AC, AQ and FD) would tend to favor the investors by restricting the earnings management practices in the firms [46–49].

As a theoretical framework, agency theory helps explain the relationships between corporate governance, earnings management and readability of the annual reports. As per agency theory, the conflicts of interest are the key issues between the managers and the shareholder because the managers have better access to the relevant and correct information regarding the financial performance of the firm than the shareholders [7]. This may result in earnings management where the managers of the business are able to alter the figures on the balance sheet in a manner that puts the business in a better light. Such practices lead to the use of complex and unclear language in annual reports, and this poses difficulties in achieving a proper evaluation of the financial health of the firm [6, 50].

Literature shows that corporate governance mechanisms as moderator played a vital. Ngatno, Apriatni [51] found that capital structure financing decisions and firm performance are positively associated. However, this relation is more substantial with the moderating effect of board size. Similarly, corporate governance quality significantly moderates the relationship between earnings management and firm performance [52]. Moreover, Salah and Jarboui [53] found the ownership concentration positively moderates the relationship between earnings management and dividend policy. Thus, the above literature highlights the importance of corporate governance as a moderator, which gives us a direction to find out either corporate governance also moderates the relationship between earnings management and readability of annual reports. Therefore, the study proposes the following hypothesis:

**Hypothesis 3:** Corporate governance mechanisms (independent board of directors, audit committee size, audit quality, female directors) moderate the relationship between earnings management and readability of annual reports.

## 3. Methodology

### 3.1. Sample

The study has taken all public listed firms in the PSX except those in the utility and financial sectors because of their different governance and capital structure. These sectors face extensive regulation: utilities are subjected to policy in order to regulate the services and its price, in the same way, financial institutions have regulatory restraints in terms of risk management and capital control [54]. The firms in utility sector usually boast of high leverage, as a result of investments on infrastructures, while financial firms retain certain ratio of capital [55]. The study used the unbalanced panel data of 250 firms and for the period of nine years from 2014 to 2022. The data is collected from mixed sources Bloomberg, DataStream, and manually from annual reports.

### 3.2. Variables measurement

The variables measurement for readability, earnings management, corporate governance mechanisms and control variables is given in the Table 1.

**Table 1:** Variables measurements.

Variables	Acronyms	Description
**Dependent Variable**		
Readability of annual reports	FI_it_	Gunning Fog Index (1952)
	FKGL_it_	Flesch-Kinciad Grade level (1975)
**Independent Variable**		
Earnings Management	ABEM_it_	Jones (1991) Model, Dechow (1995) Model, and Kothari (2005) Model
	RBEM_it_	Roychowdhury (2006) Model
	TEM_it_	Sum of ABEM and RBEM [22]
Corporate Governance		
Independent board of directors	IBD_it_	Proportion of independent board of directors
Audit Committee Size	AC_it_	Total number of members in the audit committee
Audit Quality	AQ_it_	audit firms belong to big-4 assign ‘1’, otherwise ‘0’
Female Director	*FD*_*it*_	Proportion of female board of directors
**Control Variables**		
Firm size	SIZE_it_	The natural log of total assets
Leverage	LEV_it_	Debt to total assets ratio.
Cash flow from Operations	CFO_it_	Natural log of cash flow from operations
Return on assets	ROA_it_	Net income to total assets ratio

#### 3.2.1. Measurement of the readability of annual reports

This study has used two readability indices, such as Fog Index [56], and Flesch-Kincaid grade level [57], as proxies to measure the readability of annual reports. The approach of adopting readability indices in the field of accounting and finance is consistent with the readability literature [15, 21, 50, 58]. The formula to measure Fog Index and Flesch-Kincaid grade level are stated below;

$$FI=0.4\left[(\frac{Total\;words}{Total\;sentences})+100(\frac{Complex\;words}{Total\;words})\right]$$

$$FKGL=11.8\left(\frac{Total\;syllebles}{Total\;words}\right)+0.39\left(\frac{Total\;words}{Total\;sentences}\right)-15.59$$

#### 3.2.2. Measurement of earnings management

Accrual earnings management (ABEM) and real activity earnings management (RBEM) techniques are the two primary EM approaches used by firms to manipulate earnings [13], present study have used both of the EM approaches. The present study uses three models for the measurement of ABEM such as Jones [59] model, Modifies Jones model [60], and Kothari [61] model. Moreover, study have used Roychowdhury [62], model to measure RBEM. Additionally, total earnings management (TEM) is measure using sum of ABEM and RBEM as used by Bhutto, Shaique [22].

#### 3.2.3. Measurement of corporate governance mechanisms

The present study has adopted four CG mechanisms such as independent board of directors, audit committee size, audit quality, and female board of directors.

1. **Measurement of independence board ratio:** This is measured by using the proportion of independent board of directors to the board size as used by García and Herrero [24].
2. **Measurement of audit committee size:** It is measured by the total number of members in the audit committee as used by Hoitash, Hoitash [28].
3. **Measurement of audit quality:** audit quality is measured by the dummy variable. If the firm audited by Big-4 coded ‘1’, otherwise code ‘0’ as used by Deshmukh and Zhao [40].
4. ***Measurement of female director ratio*:** This is measured total female directors divided by board size as used by García and Herrero [24].

### 3.3. The annual report cleaning procedure

The present study has adopted the procedure used by Li [15] and Loughran and McDonald [50] to generate readability index. To get the readability of financial statement disclosures study have used the following steps;

- The first step, downloaded the annual Reports which are available in PDF format.
- In the second step, the study has converted PDF to Word Docs with the help of Adobe Reader Pro. for cleaning purposes.
- In the third step, non-paragraph text (such as tables and images) was removed. Finally, it is converted into simple text files to be read by the device readability index counter software.
- In the final step, run the text files by using Perl programming language (Lingua:: EN:: Fathom).

### 3.4. Model specification

The present study has used the dynamic System generalized method of moments (GMM) econometric model, which gives robust estimates [63]. The GMM is a dynamic model that, in comparison to other models, it can manage endogeneity issue, measurement error, heteroskedasticity, unobserved individual heterogeneity, and simultaneous reverse causality [64]. Additionally, present study employed the Hausman test, which can be applied in a variety of econometric problems, to determine which model is most appropriate for our study. The results the Hausman test indicated in all the models that null hypothesis is rejected it means that study can used the system GMM for more robust results. To know the impact of earnings management on readability of annual reports following model is proposed;

$${RAR}_{it}={a_{it}+\beta }_{1}{RAR}_{it-1}+\beta_{2}{ABEM}_{it}+\sum_{j=1}^{j}\beta_{j}{Control\;Variables}_{it}^{j}\;{+\gamma }_{t}+\delta_{i}+\varepsilon_{it}$$ (1)

$${RAR}_{it}={a_{it}+\beta }_{1}{RAR}_{it-1}+\beta_{2}{RBEM}_{it}+\sum_{j=1}^{j}\beta_{j}{Control\;Variables}_{it}^{j}{+\gamma }_{t}+\delta_{i}+\varepsilon_{it}$$ (2)

$${RAR}_{it}={a_{it}+\beta }_{1}{RAR}_{it-1}+\beta_{2}{TEM}_{it}+\sum_{j=1}^{j}\beta_{j}{Control\;Variables}_{it}^{j}{+\gamma }_{t}+\delta_{i}+\varepsilon_{it}$$ (3)

In these models, i denotes the firms, and t represents the time. *RAR*_*it*_ represents the vector of different readability proxies such as Fog Index and Flesch–Kincaid; *RAR*_*it*−1_ is the lag of RAR. In model (1) and (2) *ABEM*_*it*_ and *RBEM*_*it*_ represents accrual and real EM respectively. In the model (3) *TEM*_*it*_ indicates the total earnings management (real plus accrual EM). In three models $\sum_{j=1}^{j}\beta_{j}{Control\;Variables}_{it}^{j}$ is the vector of control variables, the variable *γ*_*t*_ is time dummy variable, *δ*_*i*_ is the firms unobservable individual effects and *ε*_*it*_ shows the residual of the model. Likewise, to examine the impact corporate governance mechanisms on the readability following model is used:

$${RAR}_{it}=a_{it}+\beta_{1}{RAR}_{it-1}+\beta_{2}{IBD}_{it}{+\beta }_{3}{ACZ}_{it}{+\beta }_{4}Big4{+\beta }_{4}{FD}_{it}+\sum_{j=1}^{j}\beta_{j}{Control\;Variables}_{it}^{j}{+\gamma }_{t}+\delta_{i}+\varepsilon_{it}$$ (4)

In the above model (4) IBD_it,_ AC_it,_ Big4_it,_ and FD_it_ indicate the proportion of independent directors, audit committee size, audit quality, respectively and *ε*_*it*_ shows the residual of the model. Finally, to know the moderating impact of corporate governance mechanisms between earnings management and readability following model is used:

$${RAR}_{it}=a_{it}+\beta_{1}{RAR}_{it-1}+{\beta_{2}{ABEM}_{it}+\beta_{3}{IBD}_{it}\;{+\beta }_{4}{ACZ}_{it}{+\beta }_{5}Big4\;{+\beta }_{6}{FD}_{it}+\beta }_{7}{ABEM}_{it}*{IBD}_{it}{+\beta }_{8}AB{EM}_{it}*{ACZ}_{it}{+\beta }_{9}AB{{EM}_{it}*Big4}_{it}{+\beta }_{10}AB{EM}_{it}*{FQ}_{it}+\sum_{j=1}^{j}\beta_{j}{Control\;Variables}_{it}^{j}{+\gamma }_{t}+\delta_{i}+\varepsilon_{it}$$ (5)

$${RAR}_{it}=a_{it}+\beta_{1}{RAR}_{it-1}+{\beta_{2}{RBEM}_{it}+\beta_{3}{IBD}_{it}\;{+\beta }_{4}{ACZ}_{it}{+\beta }_{5}Big4{+\beta }_{6}{FD}_{it}+\beta }_{7}{RBEM}_{it}*{IBD}_{it}{+\beta }_{8}RB{EM}_{it}*{ACZ}_{it}{+\beta }_{9}RB{{EM}_{it}*Big4}_{it}{+\beta }_{10}RB{EM}_{it}*{FQ}_{it}+\sum_{j=1}^{j}\beta_{j}{Control\;Variables}_{it}^{j}{+\gamma }_{t}+\delta_{i}+\varepsilon_{it}$$ (6)

$${RAR}_{it}=a_{it}+\beta_{1}{RAR}_{it-1}+{\beta_{2}{TEM}_{it}+\beta_{3}{IBD}_{it}\;{+\beta }_{4}{ACZ}_{it}{+\beta }_{5}Big4{+\beta }_{6}{FD}_{it}+\beta }_{7}{TEM}_{it}*{IBD}_{it}{+\beta }_{8}RB{EM}_{it}*{ACZ}_{it}{+\beta }_{9}T{{EM}_{it}*Big4}_{it}+T{EM}_{it}*{FQ}_{it}+\sum_{j=1}^{j}\beta_{j}{Control\;Variables}_{it}^{j}{+\gamma }_{t}+\delta_{i}+\varepsilon_{it}$$ (7)

The above model (5), shown the moderation of corporate governance mechanisms between ABEM and readability of annual reports. Furthermore, model (6) represents the moderation of corporate governance mechanisms between RBEM and readability of annual reports, and model (7) indicates the moderation of corporate governance mechanisms between TEM and readability of annual reports.

## 4. Results

### 4.1. Descriptive statistics

Table 2 illustrates the summary of descriptive statistics of the variables under study. The average values of Fog Index (RAR_FI_) and, Flesch Kincaid Grade level (RAR_FKGL_) are 16.167 and 11.692 with a standard deviation of 2.326 and 1.296 respectively.

**Table 2:** Descriptive statistics.

Variables	Mean	Std. Dev.	Minimum	Maximum	Jarque-Bera (p-value)
RAR_FI_	16.167	2.326	9.3782	22.376	0.1672
RAR_FKGL_	11.692	1.296	4.842	21.108	0.2534
AC	3.497	0.529	3	8	0.2637
Big-4	0.413	0.441	0	1	0.1489
IBD	0.173	0.129	0	0.866	0.3472
FD	0.106	0.121	0	0.582	0.6727
ABEM(K)	0.022	1.138	-4.509	6.902	0.6982
ABEM(MJ)	0.049	1.102	-3.942	6.869	0.4238
ABEM(J)	0.053	0.778	-3.724	7.143	0.1493
RBEM	-0.176	0.935	-2.419	5.842	0.4749
TEM(K)	-0.154	1.145	-4.875	13.556	0.5004
TEM(MJ)	-0.127	1.225	-4.864	14.398	0.5259
TEM(J)	-0.123	1.329	-5.783	14.011	0.5515
ROA	0.063	0.121	-0.453	0.634	0.5770
Firm size	17.426	2.092	7.022	22.334	0.6026
FL	0.35	0.215	0.053	0.944	0.6809
CFO	0.062	0.178	-0.882	0.993	0.5536

The mean value of ACZ is 3.497 members, the average ratio of IBD in board size is 17.3%, and the average ratio of FD in the board size is 10.6%. The average values of ABEM (K), ABEM (MJ), and ABEM (J) are 0.022, 0.049, and 0.052, with a standard deviation of 1.138, 1.102, and 0.772, respectively. The ABEM (K) has the highest volatility among compared to the other accruals earnings management models. In addition, RBEM has average value of -0.179 with standard deviation of 0.935.

Additionally, the Jarque-Bera test is used to check the normality of the data. The null hypothesis of the Jarque-Bera test is “that the data is normally distributed”. Therefore, the p-value of all the variable in Table 2 is greater than 5% which indicates that data is normally distributed.

### 4.2. Pearson correlation matrix

The Table 3 indicates the results of Pearson correlation matrix for multicollinearity. The correlation between RAR_FI_, and RAR_FKGL_ is strong, but this is not the issue of concern as these are three different proxies for measuring. Additionally, the highest correlation among corporate governance variables is between 0.288, which is weak, and there will be no issue of multicollinearity in the model. There is a moderate correlation between Big-4 and Firm size, which is 0.426. Furthermore, the correlation among ABEM (K), ABEM (MJ), ABEM (J), RBEM, TEM (K), TEM (MJ), and TEM (J) is not an issue of concern, as these all is different proxies for measuring earnings management and have been used separately in different models.

**Table 3:** Correlation matrix.

Variables	1	2	3	4	5	6	7	8	9	10	11	12	13	14	15	16	17
**(1) RAR**_**FI**_	1.000																
**(2) RAR**_**FKGL**_	0.892	1.000															
**(3) AC**	-0.170	-0.061	1.000														
**(4) Big-4**	-0.122	-0.190	0.227	1.000													
**(5) IBD**	-0.352	-0.221	0.015	0.121	1.000												
**(6) FD**	0.012	-0.009	-0.048	-0.174	-0.152	1.000											
**(7) ABEM(K)**	0.052	0.131	-0.017	-0.092	0.006	-0.028	1.000										
**(8) ABEM(MJ)**	0.035	-0.109	-0.086	-0.064	0.004	-0.029	0.863	1.000									
**(0) ABEM(J)**	0.012	-0.032	-0.049	-0.082	-0.054	0.035	0.876	0.962	1.000								
**(10) RBEM**	-0.012	-0.019	0.032	0.043	-0.012	0.033	0.074	0.207	0.552	1.000							
**(11) TEM(K)**	0.019	0.015	-0.073	-0.025	-0.010	0.009	0.823	0.792	0.872	0.782	1.000						
**(12) TEM(MJ)**	0.022	-0.012	-0.053	-0.013	-0.011	0.045	0.796	0.721	0.892	0.758	0.902	1.000					
**(13) TEM(J)**	0.007	-0.056	-0.078	-0.036	-0.021	0.097	0.439	0.392	0.834	0.863	0.912	0.822	1.000				
**(14) ROA**	-0.072	-0.035	0.129	0.342	0.032	-0.072	-0.143	0.163	0.002	0.124	0.021	0.132	0.235	1.000			
**(15)Firm size**	-0.052	0.082	0.296	0.401	0.172	-0.102	-0.133	-0.142	-0.173	0.101	-0.072	-0.121	-0.089	0.089	1.000		
**(16) FL**	0.027	0.060	-0.242	-0.271	-0.045	0.102	0.133	0.023	0.217	0.206	0.232	0.084	0.094	-0.032	0.150	1.000	
**(17) CFO**	0.009	-0.044	0.192	0.162	-0.024	-0.017	-0.192	-0.078	-0.192	0.219	0.125	0.047	0.123	0.321	0.129	-0.192	1.000

Note: This Table 3 indicates the Pearson correlation matrix where RARFI indicates the Fog Index and RARFKGL indicates the Flesch-Kincaid Grade Level. ACZ, Big-4, IBD and FD indicate the audit committee size, audit quality, proportion of independent directors to the total directors and proportion of female directors to the total directors respectively. ABEM(K), ABEM(MJ), and ABEM(J), indicate the Kothari accrual, Modified Jones and Jones earnings management models respectively. RBEM represents the RoyChoudary real activity based earnings management. Furthermore, TEM(K) represents total earnings management which is measured by adding the ABEM(K) and RBEM. TEM(MJ) indicates total earnings management which is measured by adding the ABEM(MJ) and RBEM. TEM(J) represents total earnings management which is measured by adding the ABEM(J) and RBEM. Moreover, control variables includes: ROA is for return on assets, FL for financial leverage, Firm size for natural log of total assets and CFO represents cash flow from operations.

### 4.3. Findings

All models are generated by using the dynamic system GMM. The dynamic model can be justified as the significance of lagged dependent variable. Additionally, the AR (2) and Hansen p-values are insignificant in all models, confirming no diagnostic errors in the models. In simple, it shows that there is no issue of autocorrelation and instrument are valid.

#### 4.3.1. Impact of accruals-based earnings management on readability of annual reports

To get the comprehensive relationship between ABEM and readability of annual reports, the study has used three different proxies of ABEM as independent variables. Where ABEM(J) used in the model (1), ABEM(MJ) used in the model (2), and, ABEM(K) used in model (3). Furthermore, the dependent variable is measured by using RAR_FI_ in models (1) to (3). The empirical results of all three models in Table 4 depict that ABEM has positive effect on fog index. The empirical evidence suggests that ABEM decreases the readability of annual reports by providing complex language in the annual reports. These findings suggest that managers follow the entrenchment effect to cover up the opportunistic behavior by providing the language complexities in the annual reports. The study results are aligned with previous literature of [21] in Iran, Lo, Ramos [6] in US and Ajina et al. [2] in France.

**Table 4:** Impact of accruals-based earnings management on the readability of annual.

Variables	Expected Sign	(1)s	(2)	(3)
		RARFI	RARFI	RARFI
L.RARFI		0.2110***	0.2059***	0.2066***
ABEM(J)	+	0.1063*		
ABEM(MJ)	+		0.1670***	
ABEM(K)	+			0.08231***
ROA	-	0.0911**	-0.0458**	-0.1266***
FL	-	0.2281	-0.0754	-0.3937
Firm Size	+	0.1203***	0.0481	0.0315
CFO	-	0.2375	-0.295***	-0.2074**
Constant		12.1798***	12.8272***	12.9920***
Observations		1649	1672	1684
Arellano-Bond AR(2)		0.2944	0.2391	0.2482
Hansen Test (p-Val)		0.6372	0.4464	0.1821
Hausman test (χ2)		20.99***	13.27***	19.27***

**Note**: This table presents the System GMM results to examine the impact of ABEM RAR for Pakistani listed firms.

* p < 0.1

** p < 0.05

*** p < 0.01.

Description of variables is provided in Table 1.

#### 4.3.2. Impact of real-activity based earnings management on readability of annual reports

Table 5 represents the result regarding impact of RBEM on readability of annual reports, the findings indicate the RBEM has a significantly positive impact on Fog Index. This infers that when firms engage in real earnings management will try to produce complicated language in annual reports to hide EM. This argument is aligned with the study of Lo, Ramos [6] that firms are involved in earnings management by making less readable annual reports, which gives room to information asymmetry. In contrast, Seifzadeh, Salehi [21] argued that firms provide more readable AR when firms involve in RBEM in Tehran Market. But the study has not given the economic justification of this relationship. Furthermore, control variables are aligned with the available literature; firm size has a significantly positive impact on the RAR_FKGL_ in model (2). CFO has a significantly negative impact on the RAR_FI_ and RAR_FKGL_ in the model (1) and (2), respectively.

**Table 5:** Impact of real activity-based earning management on the readability of annual reports.

Variables	Expected	
	Sign	RAR_FI_
L.RAR_FI_		0.1977***
RBEM	**+**	0.0056***
ROA	**-**	-0.1760***
FL	**-**	-0.7761
Firm Size	**+**	0.1632***
CFO	**-**	-0.0112
Constant		13.1272***
Observations		1680
Arellano-Bond: AR(2)		0.2053
Hansen Test (p-Val)		0.4386
Hausman test (χ2)		24.36***

**Note:** This table presents the System GMM results to examine the impact of RBEM on RAR for Pakistani listed firms.

* p < 0.1

** p < 0.05

*** p < 0.01.

#### 4.3.3. Impact of total earnings management on readability of annual reports

The impact of TEM on the readability of annual reports is summarized in Table 6. The present study uses the three proxies to measure independent variable TEM such as, TEM(J) in model (1), TEM(MJ) in model (2) and TEM(K) in model (3). The results of model (2) and (3) suggest that TEM(MJ) and TEM(K) has a significantly positive impact on the RAR_FI_. Whereas, the results of model (1) suggest that TEM(J) has a positive but insignificant impact on the RAR_FI._ This suggests that firms disseminate intricate annual reports when firms have to hide EM. Zang [13] suggested that firms substitute between ABEM and RBEM on the basis of the cost associated with each earnings management strategy. In particular, firms engage in RBEM throughout the year, and ABEM is done at the year-end [13]. This infers that firms engage in both earnings management in the specific year. Therefore, the combined effect of ABEM and RBEM is useful in determining the overall influence of earnings management [12, 13].

**Table 6:** Impact of total earning management on the readability of annual reports.

Variables	Expected	1	2	3
	Sign	RAR_FI_	RAR_FI_	RAR_FI_
L.RAR_FI_		0.1423***	0.1242***	0.1162***
TEM(J)	**+**	0.0146		
TEM(MJ)	**+**		0.0372*	
TEM(K)	**+**			0.0384**
ROA	**-**	-1.2078***	-1.1486*	-1.1543**
FL	**-**	0.4598	0.1720	-0.4713
Firm Size	**+**	0.0628*	0.0545	0.0948**
CFO	**-**	-0.2447	-0.5859**	-0.3938
Constant		12.7846***	13.1532***	12.9234***
Observations		1661	1673	1697
Arellano-Bond: AR(2)		0.3467	0.3165	0.2901
Hansen Test (p-Val)		0.5274	0.5522	0.4872
Hausman test (χ2)		17.34***	16.37***	29.77***

**Note:** This table presents the System GMM results to examine the impact total EM on the RAR for Pakistani listed firms.

* p < 0.1

** p < 0.05

*** p < 0.01.

These results are especially pertinent to the context of Pakistan given the typically lower educational levels of investors and comparatively lesser regulatory scrutiny as compared to highly developed countries [65]. Using such complicated language may work in the favor of managers due to the obscurity it brings to the earnings management activities in situations where some investors may not be fully knowledgeable, an issue that is very relevant in the context of Pakistan as its market for both finance and corporate governance continues to evolve [66].

#### 4.3.4. Impact of corporate governance mechanisms on the readability of annual reports

Table 7 illustrates the results of system GMM to investigate the relationship between RAR_FI_ and RAR_FKGL_ as dependent variables and corporate governance mechanisms as independent variables along with control variables. The results indicate that corporate governance mechanisms such as ACZ, Big-4, IBD, and FD negatively impact RAR_FI_ and RAR_FKGL_. It demonstrates an increase in any corporate governance mechanisms (i.e., ACZ, Big-4, IBD, and FD) will decrease the RAR_FI_ and RAR_FKGL_. In short, these four corporate governance mechanisms may improve the RAR in the Pakistani market.

**Table 7:** The impact of CG mechanisms on readability of annual reports.

Variables	Expected	(1)
	Sign	RAR_FI_
L. RAR_FI_		0.1488***
AC	**-**	-0.4328***
AQ	**-**	-0.2845***
IBD	**-**	-1.9483***
FD	**-**	-2.2532***
ROA	**-**	-0.1193
FL	**-**	-0.7443***
Firm Size	**+**	0.0382
CFO	**-**	-0.4021**
Constant		14.9487***
Observations		1679
Arellano-Bond: AR(2)		0.2834
Hansen Test (p-Val)		0.1726
Hausman test (χ2)		21.42***

**Note:** This table presents the System GMM results to examine the impact of CG mechanisms on the RAR for Pakistani listed firms.

* p < 0.1

** p < 0.05

*** p < 0.01.

The AC plays a significant role in disseminating transparent information by improving the readability. The result is consistent with the available literature that ACZ effectively provides transparent financial reporting [28, 29]. Similarly, Big-4 may enhance the readability by providing easy-to-read annual reports. This argument is aligned with the study of Shahzad, Rehman [33] and Clinch, Stokes [39]. Moreover, when the number of IBD increases, there will be more monitoring and direct control on providing the ease to understand information in the annual reports. The support of this argument can be drawn from the study of, and Tessema [25]. The FD in the board improves readability of annual reports to facilitate the stakeholders. This results are aligned with study of Ginesti, Drago [42].

Additionally, these findings have important implications in the Pakistani setting as they emphasize that better corporate governance can result in the enhancement of financial reporting quality. Considering the limitations of high quality governance practices in Pakistan including cultural and regulatory factors, these result call for change in policies to more profoundly build up the governance structures to make the financial report more readable and transparent [66].

#### 4.3.5. Moderating impact of corporate governance mechanisms between ABEM and readability of annual reports

Table 8 illustrates the moderating effect of corporate governance mechanisms between the relationship ABEM and readability of annual reports, including the interaction of each corporate governance mechanism with ABEM to observe the changing impact of ABEM on readability of annual reports. The findings indicate all the ABEM models have positive effect on RAR_FI,_ which is consistent with Table 4. While corporate governance mechanisms, ACZ, Big-4, IBD, and FD have a negative impact on RAR_FI_, which is also consistent with Table 7. With respect to the moderating relationship of corporate governance variables in Table 8, the positive impact of ABEM on RAR_FI_ in all three models changed when it is moderated with corporate governance variables. Interaction of ACZ and FD with ABEM in model (1) to (3) significantly moderates the relationship as sign changed from positive to negative, means that audit committee and female directors are effective in reducing the ABEM by promoting ease to understand the information in the annual reports. Moreover, IBD significantly moderates the relationship between ABEM with RAR_FI_ in model (1) and (2) as the coefficient sign of ABEM is changed from positive to negative. Further, Big-4 moderates the relationship in model (1) only. The support of moderating relationship of corporate governance can be drawn from the study of Jane and Xueji [67] that the inclusion of corporate governance (board independence and board activeness) as moderator changed the coefficient sign from negative to positive between internationalization and firm performance. Similarly, the moderating role of corporate governance also supported by the study of Boachie & Mensah [52].

**Table 8:** Moderating impact of CG mechanisms between ABEM and RAR.

Variables	Expected	(1)	(2)	(3)
	Sign	RAR_FI_	RAR_FI_	RAR_FI_
L.RAR_FI_		0.3923***	0.2162***	0.1925***
ABEM(J)	+	0.5672***		
ABEM(MJ)	+		0.6627***	
ABEM(K)	+			0.6329***
AC	-	-0.3587***	-0.6940***	-0.3570***
AQ	-	-0.4472***	-0.2836***	-0.3923***
IBD	-	-1.6973***	-1.2563***	-1.6421***
FD	-	-0.2644	-0.5256**	-0.6027***
ABEM(J)*AC	-	-0.2629***		
ABEM(J)*AQ	-	-0.1977**		
ABEM(J)*IBD	-	-0.0028		
ABEM(J)*FD	-	-0.5173**		
ABEM(MJ)*AC	-		-0.1829***	
ABEM(MJ)*AQ	-		-0.0358	
ABEM(MJ)*IBD	-		-0.3927**	
ABEM(MJ)*FD	-		-0.1782***	
ABEM(K)*AC	-			-0.1928***
ABEM(K)*AQ	-			-0.0826*
ABEM(K)*IBD	-			-0.5478***
ABEM(K)*FD	-			-0.2742***
ROA	-	-0.8324***	-0.1290	-0.3849***
FL	-	-0.0729	-0.3027**	-0.7266***
Firm Size	+	0.0532***	0.0728***	0.06520***
CFO	-	0.1110	0.1629	0.1525
Constant		13.5232***	13.2928***	11.7201***
Observations		1673	1682	1690
Arellano-Bond: AR(2)		0.3533	0.2792	0.4932
Hansen Test (p-Val)		0.4542	0.3321	0.1920
Hausman test (χ2)		25.27***	34.48***	30.65***

**Note:** This table presents the System GMM results to examine the moderating impact CG mechanisms between accruals EM and RAR for Pakistani listed firms.

* p < 0.1

** p < 0.05

*** p < 0.01.

#### 4.3.6. Moderating impact of corporate governance mechanisms between RBEM and readability of annual reports

Table 9 demonstrates the moderating impact of corporate governance on the relationship between RBEM and readability of annual reports by including the interaction of each corporate governance mechanism. The results indicates that the positive and significant coefficient of RBEM moderated when it has interacted with ACZ, Big-4 and, IBD. Therefore, it can be argued that audit committee size, audit quality and independent directors, strengthen the governance system of the firm by limiting the RBEM and providing more readable annual reports. This result is consistent with the study of Hastuti, Ghozali [68].

**Table 9:** Moderating impact of CG mechanisms between RBEM and RAR.

Variables	Expected	(1)
	sign	RAR_FI_
L.RAR_FI_		0.2016***
RBEM	+	0.7729***
AC	-	-0.8232***
AQ	-	-0.7183*
IBD	-	-2.8586***
FB	-	-1.927***
RBEM*AC	-	-0.0958*
RBEM*AQ	-	-0.4973***
RBEM*IBD	-	-0.7892*
RBEM*FD	-	0.2390***
ROA	-	-0.7269*
FL	-	0.2975
Firm Size	+	0.1021*
CFO	-	-0.6589
Constant		14. 9296***
Observations		1678
Arellano-Bond: AR(2)		0.4076
Hansen Test (p-Val)		0.2183
Hausman test (χ2)		37.52***

**Note:** This table presents the System GMM results to examine the moderating impact CG mechanisms between RBEM and RAR for Pakistani listed firms.

* p < 0.1

** p < 0.05

*** p < 0.01.

#### 4.3.7. Moderating impact of corporate governance mechanisms between TEM and RAR

Table 10 exhibits the impact of TEM with the RAR along with moderating impact of ACZ, Big-4 IBD, and FD between them. With respect to the moderating relationship of corporate governance variables, the positive impact of TEM on RAR_FI_ in all three models changed when it is moderated with corporate governance variables. The Big-4 has negatively significant moderation on RAR_FI_ in three models. Whereas, the ACS has moderating impact on RAR_FI_ in model (2) and (3). Moreover, IBD has significant moderating impact in model (1) and (3), while FD has no moderating impact between TEM and readability of annual reports. The results of the study complement that firms use less readable annual reports to cover up TEM, which can be reduced through implementing an effective corporate governance structure.

**Table 10:** Moderating impact of CG mechanisms between TEM and RAR.

Variables	Expected	(1)	(2)	(3)
	Sign	RAR_FI_	RAR_FI_	RAR_FI_
L.RAR_FI_		0.1689***	0.1267***	0.1728***
TEM(J)	+	0.2032**		
TEM(MJ)			0.3920**	
TEM(K)				0.3356***
AC	-	-0.2869***	-0.4462***	-0.3920***
AQ	-	-0.3832***	-0.1938***	-0.2459***
IBD	-	-1.9624***	-1.7622***	-1.928***
FD	-	-0.7284***	-0.1926*	-0.8238**
TEM(J)*AC	-	-0.1458		
TEM(J)*AQ	-	-0.2845*		
TEM(J)*IBD	-	-0.3263**		
TEM(J)*FD	-	0.1128**		
TEM(MJ)*AC	-		-0.0639**	
TEM(MJ)*AQ	-		-0.0718*	
TEM(MJ)*IBD	-		-0.3953*	
TEM(MJ)*FD	-		0.1729*	
TEM(K)*AC	-			-0.1421***
TEM(K)*AQ	-			-0.0827*
TEM(K)*IBD	-			-0.3428*
TEM(K)*FD	-			0.2322
ROA	-	0.0826	-0.2321*	-0.3225**
FL	-	-0.7284**	-0.4253*	-0.4216**
Firm Size	+	0.0927*	0.0853***	0.0273**
CFO	-	0.3135	0.6342	0.2526
Constant		13.2324***	14.3242***	14.9262***
Observations		1663	1678	1692
Arellano-Bond: AR(2)		0.2971	0.5624	0.2629
Hansen Test (p-Val)		0.4026	0.3802	0.2232
Hausman test (χ2)		23.99***	34.27***	31.72***

**Note:** This table presents the System GMM results to examine the moderating impact CG mechanisms between RBEM and RAR for Pakistani listed firms. Standard errors in brackets.

* p < 0.1

** p < 0.05

*** p < 0.

In the context of Pakistan, the result therefore call for reforms that seek to enhance effective implementation of proper corporate governance standards so as to minimize the opportunities for earnings management and ensure appropriate and complete reports to stakeholders are provided.

#### 4.3.8. Robustness check

To further check the reliability of our results, the present study has used another readability index such as Flesch-Kincaid grade level. The findings generally conform to our earlier results across all the models. The robustness check results are provided in the S1 Appendix. The positive coefficient of earnings management (ABEM, RBEM and TEM) in Tables XI-XIII in S1 Appendix indicates that firms provides less readable annual reports to cover EM. Furthermore, Table XIV in S1 Appendix provides the results that corporate governance mechanisms (ACZ, Big-4, IBD, and FD) are effective in providing more readable annual reports. In addition to the moderating role of corporate governance is provided in Tables XV-XVII in S1 Appendix. The results shows the corporate governance mechanisms (ACZ, Big-4, IBD, and FD) significantly moderates the relationship between earnings management (ABEM, RBEM and TEM) and readability of annual reports.

## 5. Conclusion

This study examines the impact of corporate governance mechanisms and earnings management (accrual, real and total) on readability of annual reports by using non-financial firms listed in PSX for the period of 2014–2022. Furthermore, this study also examined the moderating role of corporate governance mechanism between earnings management and readability of annual reports. The present study found that firms that engage in earnings management (real, accruals or total) provide more language complexities to make annual reports less readable. The firms intentionally make annual reports difficult to understand to overshadow managers’ opportunistic behavior. The study’s findings support the view that audit committee size, audit quality, the proportion of independent directors, and female directors can be beneficial for corporate governance mechanisms to disseminate easy-to-read annual reports. Furthermore, the study’s findings support the assumption that corporate governance mechanisms significantly moderates the relationship between earnings management (accruals, real or total) and readability of annual reports. The results of the study may recommend to the Security Exchange Commission that authorities may take actions to enhance the readability by compelling firms to use the simple and understandable language and provide the relevant information in the annual reports. In addition, it may enable regulatory authorities to look for the readability of annual reports assessment by giving greater importance to the transparency of the disclosed information. The investors’ main source of information is the annual report which helps them to make decisions. Hence, investors may not just relay on information provided in annual reports for making any decisions.

There are certain limitations to the current study that indicate future research options. Firstly, the present study is confined to Pakistan’s non-financial sectors, the findings can be applied to the non-financial sectors of other countries with similar regulatory environments. The present study have used two indices of readability (FOG and FLESCH-KINCAID), while future studies may consider other readability indices. The present study has used four corporate governance mechanisms, while future studies may consider the other corporate governance variables such as, family ownership, institutional ownership, managerial ownership, foreign BODs, and political connection.

## Supporting information

S1 Appendix (DOCX)

S1 Data (XLSX)
