# Supplementary material for: Does corporate governance mechanism deter earnings management and enhance readability of annual reports?
Source: PLoS One. 2025 Feb 14;20(2):e0311543. doi: 10.1371/journal.pone.0311543 (PMC11828418; doi:10.1371/journal.pone.0311543)
Supplement: S1 Appendix — (DOCX) [file pone.0311543.s001.docx]

**Appendix**

**Table XI**: Impact of ABEM on RAR

| **Variables** | **Expected** | **(1)** | **(2)** | **(3)** |
| --- | --- | --- | --- | --- |
|  | **Sign** | **RAR_FKGL_** | **RAR_FKGL_** | **RAR_FKGL_** |
| L.RAR_FKGL_ |  | 0.2176*** | 0.1782*** | 0.1928*** |
| ABEM(J) |  | 0.0218 |  |  |
| ABEM(MJ) |  |  | 0.0242** |  |
| ABEM(K) | **+** |  |  | 0.0635** |
| ROA | **-** | -1.7236** | -1.3723*** | 0.4252 |
| FL | **-** | -0.2722 | 0.1332 | 0.0532 |
| Firm Size | **+** | 0.0352** | 0.0421*** | 0.0937* |
| CFO | **-** | -0.0532 | 0.7422 | -0.4629* |
| Constant |  | 8.1314*** | 8.2426*** | 9.0272** |
| Observations |  | 1655 | 1671 | 1686 |
| Arellano-Bond: AR(2) |  | 0.8361 | 0.4202 | 0.2982 |
| Hansen Test (p-Val) |  | 0.6247 | 0.7293 | 0.2638 |
| **Note:** This table presents the System GMM results to examine the impact of accrual-based EM on the readability of annual reports for Pakistani listed firms.* p < 0.1, ** p < 0.05, *** p < 0.01. | | | | |

**Table XII:** Impact of RBEM on RAR

| **Variables** | **Expected** | **(1)** |
| --- | --- | --- |
|  | **Sign** | **RAR_FKGL_** |
| L.RAR_FKGL_ |  | 0.1672*** |
| RBEM | **+** | 0.0973* |
| ROA | **-** | -0.01825 |
| FL | **-** | -0.023 |
| Firm Size | **+** | 0.1937** |
| CFO | **-** | -0.0203*** |
| Constant |  | 8.3242*** |
| Observations |  | 1686 |
| Arellano-Bond: AR(2) |  | 0.2932 |
| Hansen Test (p-Val) |  | 0.3382 |
| Note: This table presents the System GMM results to examine the impact of real-based EM on the readability of annual reports for Pakistani listed firms. * p < 0.1, ** p < 0.05, *** p < 0.01. | | |

| **Table XIII: Impact of TEM on RAR** | | | | |
| --- | --- | --- | --- | --- |
| **Variables** | **Expected** | **(1)** | **(2)** | **(3)** |
|  | **Sign** | **RAR_FKGL_** | **RAR_FKGL_** | **RAR_FKGL_** |
| L.RAR_FKGL_ |  | 0.1677*** | 0.1749*** | 0.2281*** |
| TEM(J) | + | 0.0131 |  |  |
| TEM(MJ) | + |  | 0.0343** |  |
| TEM(K) | + |  |  | 0.0342** |
| ROA | - | -12422** | -1.2532* | 1.0222 |
| FL | - | -0.1034 | -0.0837 | -0.0939 |
| Firm Size | + | 0.0852*** | 0.0987*** | 0.0729*** |
| CFO | - | -0.3230 | -0.74292** | -0.97345*** |
| Constant |  | 8.9623*** | 9.7356*** | 9.2432*** |
| Observations |  | 1655 | 1675 | 1696 |
| Arellano-Bond: AR(2) |  | 0.2038 | 0.3622 | 0.3485 |
| Hansen Test (p-Val) |  | 0.4520 | 0.7462 | 0.4937 |
| Note: This table presents the System GMM results to examine the impact of total EM on the readability of annual reports for Pakistani listed firms. * p < 0.1, ** p < 0.05, *** p < 0.01. | | | | |

| **Table XIV: The impact of CG mechanisms on RAR** | | |
| --- | --- | --- |
|  | **Expected** | (1) |
|  | **Sign** | **RAR_FKGL_** |
| L. RAR_FKGL_ |  | 0.1403*** [0.019] |
| AC | **-** | -0.1832*** [0.044] |
| AQ | **-** | -0.1424** [0.057] |
| IBD | **-** | -1.1905*** [0.229] |
| FD | **-** | -0.9954*** [0.359] |
| ROA | **-** | -0.0551 [0.132] |
| FL | **-** | -0.0399 [0.132] |
| Firm Size | **+** | 0.0771*** [0.017] |
| CFO | **-** | -0.2568* [0.146] |
| Constant |  | 9.5103*** [0.351] |
| Observations |  | 757 |
| Arellano-Bond: AR(2) |  | 0.2406 |
| Hansen Test (p-Val) |  | 0.3565 |
| Note: This table presents the System GMM results to examine the impact of CG mechanisms on the readability of annual reports for Pakistani listed firms. * p < 0.1, ** p < 0.05, *** p < 0.01. | | |

| **Table XV: Moderating impact of CG mechanisms between ABEM and RAR** | | | | |
| --- | --- | --- | --- | --- |
| **Variables** | **Expected** | **(1)** | **(2)** | **(3)** |
|  | **Sign** | **RAR_FKGL_** | **RAR_FKGL_** | **RAR_FKGL_** |
| L.RAR_FI_ |  | 0.1482*** | 0.1327*** | 0.1155*** |
| ABEM(J) | + | 0.5725*** |  |  |
| ABEM(MJ) |  |  | 0.5325*** |  |
| ABEM(K) |  |  |  | 0.4529*** |
| AC | - | -0.1387*** | -0.1143*** | -0.1328*** |
| AQ | - | -0.3472*** | -0.3392*** | -0.2789** |
| IBD | - | -1.9213*** | -1.5332* | -1.7282*** |
| FD | - | -0.3763*** | 0.2987* | -0.3795** |
| ABEM(J)*AC | - | -0.3332*** |  |  |
| ABEM(J)*AQ | - | -0.2583** |  |  |
| ABEM(J)*IBD | - | -0.3262** |  |  |
| ABEM(J)*FD | - | -0.3328*** |  |  |
| ABEM(MJ)*AC | - |  | -0.0832*** |  |
| ABEM(MJ)*AQ | - |  | -0.1863** |  |
| ABEM(MJ)*IBD | - |  | -0.4424*** |  |
| ABEM(MJ)*FD | - |  | -0.1825*** |  |
| ABEM(K)*AC | - |  |  | -0.1286*** |
| ABEM(K)*AQ | - |  |  | -0.1826* |
| ABEM(K)*IBD | - |  |  | -0.5324*** |
| ABEM(K)*FD | - |  |  | -0.1238* |
| ROA | - | -0.1313** | -0.3825* | 0.1936 |
| FL | - | -0.0623 | -0.0836 | 0.020 |
| Firm Size | + | 0.0434*** | 0.0647*** | 0.0536** |
| CFO | - | -0.3364 | 0.0259 | -0.1639 |
| Constant |  | 10.5324*** | 9.9362*** | 10.0291*** [0.505] |
| Observations | | 1655 | 1669 | 1682 |
| Arellano-Bond: AR(2) | | 0.3463 | 0.2387 | 0.5324 |
| Hansen Test (p-Val) | | 0.5467 | 0.3824 | 0.4279 |
| Note: This table presents the System GMM results to examine the moderating impact CG mechanisms between accruals EM and readability of annual reports for Pakistani listed firms. * p < 0.1, ** p < 0.05, *** p < 0.01. | | | | |

| **Table XVI: Moderating impact of CG mechanisms between RBEM and RAR** | | |
| --- | --- | --- |
| **Variables** | **Expected** | **(1)** |
|  | **sign** | **RAR_FKGL_** |
| L. RAR_FKGL_ |  | 0.1967*** |
| RBEM | + | 0.2152* |
| AC | - | -0.1326** |
| AQ | - | -0.2563** |
| IBD | - | -1.2862*** |
| FB | - | -0.2198* |
| RBEM*AC | - | -0.0365 |
| RBEM*AQ | - | -0.2173*** |
| RBEM*IBD | - | -0.4529** |
| RBEM*FD | - | 0.1365* |
| ROA | - | -0.0529* |
| FL | - | 0.1283 |
| Firm Size | + | 0.0637** |
| CFO | - | -0.0124* |
| Constant |  | 10.1378*** |
| Observations |  | 1682 |
| Arellano-Bond: AR(2) |  | 0.2853 |
| Hansen Test (p-Val) |  | 0.2963 |
| Note: This table presents the System GMM results to examine the moderating impact CG mechanisms between real EM and readability of annual reports for Pakistani listed firms. * p < 0.1, ** p < 0.05, *** p < 0.01. | | |

| **Table XVII: Moderating impact of CG mechanisms between TEM and RAR** | | | | |
| --- | --- | --- | --- | --- |
| **Variables** | **Expected** | **1** | **2** | **3** |
|  | **Sign** | **RAR_FKGL_** | **RAR_FKGL_** | **RAR_FKGL_** |
| L.RAR_FKGL_ |  | 0.1738*** | 0.1638*** | 0.1418*** |
| TEM(J) | + | 0.2751** |  |  |
| TEM(MJ) |  |  | 0.3025*** |  |
| TEM(K) |  |  |  | 0.2530*** |
| AC | - | -0.2638*** | -0.3382*** | -0.1728*** |
| AQ | - | -0.3428*** | -0.2842*** | -0.3427*** |
| IBD | - | -1.6483*** | -1.8326*** | -1.3282*** |
| FD | - | -0.5263** | -0.2954* | 0.4242* |
| TEM(J)*AC | - | -0.0274* |  |  |
| TEM(J)* AQ | - | -0.1035** |  |  |
| TEM(J)*IBD | - | -0.0642* |  |  |
| TEM(J)*FD | - | 0.0653 |  |  |
| TEM(MJ)*AC -  TEM(MJ)*AQ -  TEM(MJ)*IBD -  TEM(MJ)*FD - | |  | -0.1835*** |  |
|  |  |  | -0.0925* |  |
|  |  |  | 0.2292* |  |
|  |  |  | 0.1428* |  |
| TEM(K)*AC | - |  |  | -0.0343*** |
| TEM(K)*AQ | - |  |  | -0.0843*** |
| TEM(K)*IBD | - |  |  | -0.0325 |
| TEM(K)*FD | - |  |  | -0.1042* |
| ROA | - | -0.8362** | 0.2730** | -0.3263*** |
| FL | - | 0.0693 | 0.0983 | 0.0847 |
| Firm Size | + | 0.1738*** | 0.1342*** | 0.1952*** |
| CFO | - | 0.2046 | 0.1035 | 0.1173 |
| Constant |  | 9.3729*** | 8.5472*** | 9.9545*** |
| Observations |  | 1677 | 1678 | 1689 |
| Arellano-Bond: AR(2) |  | 0.2324 | 0.4253 | 0.2422 |
| Hansen Test (p-Val) |  | 0.3234 | 0.4362 | 0.4683 |
| Note: This table presents the System GMM results to examine the moderating impact CG mechanisms between TEM and readability of annual reports for Pakistani listed firms. * p < 0.1, ** p < 0.05, *** p < 0.01. | | | | |
